# Telepsychology and digital mental health communication: an exploratory mixed-methods study of tikTok content

**DOI:** 10.3389/fdgth.2026.1900544

**Published:** 2026-09-15

**Authors:** Andrea M. Carrero, Jobana F. Agredo, Lucía Catalina Martínez Ruiz, José A. González

**Affiliations:** 1 Psychology Program, School of Social Sciences, Arts and Humanities, Universidad Nacional Abierta y a Distancia (UNAD), Ibagué, Colombia; 2 Psychology Program, School of Social Sciences, Arts and Humanities, Universidad Nacional Abierta y a Distancia (UNAD), Bogotá, Colombia; 3 Psychology Program, School of Social Sciences, Arts and Humanities, Universidad Nacional Abierta y a Distancia (UNAD), Cúcuta, Colombia; 4 Department of Mathematics, Physics and Statistics, Faculty of Basic Sciences, Universidad Católica del Maule, Talca, Chile

**Keywords:** digital health literacy, digital mental health, discursive transposition, health communication, mixed-methods research, social media, telepsychology, tikTok

## Abstract

**Background:**

Telepsychology consolidated as a mainstream mental health care modality following the COVID-19 pandemic, supported by evidence of clinical efficacy comparable to in-person therapy and a recognized potential to democratize access to care. Against this evolving clinical landscape, social media platforms have simultaneously emerged as major environments for the public circulation of psychological knowledge.

**Objective:**

To explore the relationship between scientific evidence on telepsychology and its discursive representation in highly viewed Spanish-language TikTok content, and to identify implications for digital mental health literacy and professional communication practices.

**Methods:**

An exploratory mixed-methods design with qualitative emphasis integrated a scoping review (PRISMA-ScR) across Scopus, Web of Science, and PubMed (2020–2025; *n* = 39 articles) and an inductive thematic analysis of TikTok content (*N* = 50 videos; 1,286,360 total views). Searches were conducted from a newly created TikTok account with no prior interaction history, configured in Colombia, to reduce algorithmic personalization bias. Three exploratory corpus-descriptive indicators—the Telepsychology Gap Index (TGI), Digital Telepsychology Legitimacy Index (DTLI), and Simplified Circulation Index (SCI)—were computed as heuristic comparators.

**Results:**

The scientific literature consistently supported the clinical viability and democratizing potential of telepsychology. Within the TikTok corpus, 64% of content featured simplified definitions or subjectivist experiential narratives with minimal reference to ethical or regulatory frameworks. Corpus-descriptive indicators revealed a substantial gap between scientific representation and digital circulation (TGI = 0.80), limited presence of clinical foundations in digital discourse (DTLI = 0.36), and a predominance of simplified over technically grounded content (SCI = 1.78).

**Conclusions:**

Within the analyzed corpus, telepsychology undergoes a process of digital discursive reinterpretation in which communicative accessibility and immediate subjective experience displace clinical precision as central evaluative criteria. These findings underscore the need to strengthen digital mental health literacy and professionals' evidence-based communication skills online. Given the exploratory design, findings are specific to a non-probabilistic Spanish-language TikTok corpus and are not generalizable prevalence estimates. Future research should extend this agenda through larger multilingual samples, additional platforms, computational social science approaches, and longitudinal designs examining how digital representations of telepsychology shape users' expectations and engagement with digital mental health care.

## 1 Introduction

Telepsychology initially emerged as a remote service delivery alternative for geographically underserved populations. Its expansion accelerated dramatically during the COVID-19 pandemic, consolidating remote psychological care as a clinically accepted and institutionally recognized modality of digital mental health service delivery (1, 2). Across healthcare systems worldwide, the use of digital technologies to ensure continuity of psychological services increased from approximately 7% to 85% of practice within a matter of months (3). According to the World Health Organization, disruptions to mental health services affected vulnerable populations in over 130 countries (4), reinforcing the urgent need for accessible remote care alternatives.

Within this context, telepsychology demonstrated important logistical and clinical advantages: improved accessibility, continuity of care, scheduling flexibility, reduced geographic barriers, and expanded service reach among underserved populations (5–8). The American Psychological Association defines telepsychology as the provision of psychological services using information and communication technologies (ICTs), including videoconferencing, digital platforms, mobile applications, and other remote communication tools (9). Beyond its technological dimension, telepsychology encompasses ethical, legal, technical, and clinical considerations associated with professional psychological practice in digital environments (10, 11).

Current evidence supports the effectiveness of telepsychology across multiple intervention contexts—anxiety disorders, depressive symptomatology, psychosocial support, and psychological follow-up—with therapeutic outcomes and patient satisfaction levels comparable to face-to-face care when structured evidence-based approaches are employed (12–15). Nevertheless, rapid expansion has generated important debates regarding professional competencies, therapeutic alliance adaptation, confidentiality, digital inequality, and the absence of standardized international regulatory frameworks (16–20). To interpret this relationship more precisely, the following theoretical framework brings together three complementary lenses: discursive transposition, digital information ecosystems, and digital mental health literacy. These perspectives provide the conceptual basis for understanding how telepsychology may be transformed when it circulates through high-visibility platform environments such as TikTok.

### 1.1 Tiktok as an environment of psychological knowledge circulation

Concurrently, social media platforms increasingly function as influential environments for the circulation, reinterpretation, and simplification of mental health-related information. Among these platforms, TikTok has achieved exceptional visibility through its short-form audiovisual format, engagement-driven recommendation algorithms, and participatory communication dynamics (21–23). Mental health content on TikTok has expanded rapidly, particularly among younger Spanish-speaking users seeking accessible explanations, emotional validation, and self-help narratives (Naslund et al., 2020; Zenone et al., 2023; McCashin & Murphy, 2023).

Within algorithmically mediated environments, communication dynamics frequently prioritize immediacy, emotional resonance, and audience engagement over technical precision and scientific contextualization (24–26). From the perspective of discursive transposition, specialized knowledge is transformed into communicatively functional versions adapted to broader audiences and novel circulation environments (27). These transformations may facilitate mass dissemination while simultaneously reducing conceptual complexity and clinical nuance (23, 28). In digital mental health contexts, such simplification may contribute to unrealistic therapeutic expectations, decontextualized interpretations of clinical terminology, or misunderstandings regarding professional standards—though they should not automatically be equated with deliberate misinformation (23, 29).

Against this backdrop, the present study aims to explore the relationship between scientific evidence on telepsychology and its discursive representation within highly viewed TikTok content in Spanish. Specifically, the study examines how platform-mediated communication dynamics may contribute to the social reinterpretation and digital circulation of telepsychology within contemporary digital mental health ecosystems.

### 1.2 Theoretical framework

#### 1.2.1 Discursive transposition and the social circulation of psychological knowledge

Discursive transposition refers to the transformation of specialized knowledge into communicatively accessible forms intended for broader social circulation (27, 30). In health communication contexts, this process involves selection, simplification, reinterpretation, and recontextualization of expert knowledge to facilitate public understanding. Although transposition may contribute positively to accessibility, it may simultaneously reduce conceptual precision and clinical contextualization. In social media environments optimized for brevity, emotional resonance, and algorithmic visibility, these transformations are intensified (24, 28).

The circulation of psychological knowledge within digital platforms no longer depends exclusively on institutional or academic actors. Users, influencers, content creators, and recommendation algorithms increasingly participate in the production, amplification, and reinterpretation of mental health narratives (21, 24). From this perspective, digital platforms function not merely as neutral dissemination channels but as active communicative environments that shape the forms through which psychological knowledge is socially interpreted and emotionally appropriated.

#### 1.2.2 Digital information ecosystems and mental health communication

Contemporary digital ecosystems are characterized by accelerated information flows, algorithmically mediated visibility structures, and participatory communication logics. The concept of infodemic—introduced by the World Health Organization to describe the overabundance of information that impedes access to reliable guidance (31)—acquired particular relevance during the COVID-19 pandemic and remains applicable to ongoing digital mental health discourse dynamics. Mental health content has become one of the most rapidly expanding thematic categories across social media platforms (22, 25). TikTok's recommendation algorithm privileges highly engaging content capable of generating rapid interaction and emotional identification, reinforcing communication dynamics centred on accessibility, relatability, and immediacy regardless of technical content quality.

#### 1.2.3 Cultural influences in digital mental health communication

Digital mental health content does not circulate in a culturally neutral environment. Platform-mediated communication is shaped by language, local norms of emotional expression, collective understandings of psychological distress, and culturally specific expectations regarding help-seeking, authority, and professional legitimacy. In this sense, the meanings attached to telepsychology in social media cannot be explained solely by platform affordances; they are also filtered through the cultural frameworks within which users interpret care, expertise, and mental health narratives.

This cultural dimension is especially relevant in Spanish-language digital ecosystems, where mental health discourse may combine clinical terminology with everyday idioms, relational values, and locally meaningful forms of emotional validation. Cross-cultural research has shown that the relationship between social media use and mental health outcomes can vary according to cultural background and the ways digital practices are measured and interpreted. Accordingly, the representation of telepsychology on TikTok should be understood not only as a product of algorithmic visibility and communicative simplification, but also as a culturally situated form of discursive adaptation that reflects how mental health knowledge is socially appropriated in specific linguistic and regional contexts.

#### 1.2.4 Digital mental health literacy

Digital health literacy refers to the ability to search for, understand, critically evaluate, and appropriately apply health-related information obtained through digital environments (32). In mental health contexts, this construct encompasses the capacity to distinguish evidence-based information from subjective testimony, commercialized self-help discourse, and decontextualized psychological explanations (26, 33). As telepsychology becomes increasingly normalized within digital culture, users frequently construct their expectations of online therapy through content encountered on social media prior to initiating professional contact (23, 29). Consequently, communication competencies in digital mental health environments acquire growing relevance—not only for public education but also for responsible professional practice and training programme design (7, 18).

## 2 Materials and methods

### 2.1 Study design

The study employed an exploratory mixed-methods design with qualitative emphasis, integrating two complementary phases: (1) a scoping review of the scientific literature and (2) an inductive thematic analysis of TikTok discourse. The quantitative component—represented by three corpus-descriptive heuristic indicators—is subordinate to the qualitative interpretive logic and does not constitute the dominant analytical component. This decision responded to the nature of the TikTok corpus: non-probabilistic, small, discursive, and algorithmically mediated—conditions that favour an exploratory interpretive approach over inferential modelling. The study did not seek statistical representativeness but rather analytical exploration of how telepsychology is communicatively reframed within digital environments characterized by rapid information circulation and engagement-oriented platform structures. The study was designed and reported in accordance with current recommendations for transparent reporting of qualitative and mixed-methods research whenever applicable.

### 2.2 Scoping review

A scoping review was conducted following the methodological guidance of Peters et al. (34) and reported according to the PRISMA Extension for Scoping Reviews (PRISMA-ScR) framework (35). The review protocol was prospectively registered on the Open Science Framework (OSF) prior to data extraction. The research question was structured using the Population, Concept, and Context (PCC) framework: Population—adults receiving or delivering psychological services; Concept—telepsychology as a modality of mental health care; Context—digital environments, 2020–2025, with reference to professional, ethical, or clinical frameworks.

Searches were performed across Scopus, Web of Science, and PubMed using controlled descriptors and free-text terms combined with Boolean operators: (telepsychology OR telehealth OR “online counseling” OR “digital mental health” OR telepsicología) AND (mental health OR psychotherapy OR clinical psychology), applied across titles, abstracts, and keywords. Consistent with the exploratory purpose of scoping reviews, the design did not include formal risk-of-bias appraisal (34).

Inclusion criteria comprised articles published between January 2020 and February 2025, in English or Spanish, available in full text, and explicitly addressing telepsychology practice, implementation, effectiveness, ethics, accessibility, or professional competencies. Editorials, conference abstracts, dissertations, non-peer-reviewed publications, duplicated records, and studies not directly related to telepsychology were excluded. Selection followed four stages (identification, duplicate removal, title/abstract screening, full-text assessment) conducted independently by two researchers; discrepancies were resolved by consensus. The final sample comprised 39 eligible publications. The complete selection flow is presented in Figure 1 (PRISMA-ScR). The full extraction matrix of all 39 included studies is available in the OSF repository.

**Figure 1:**
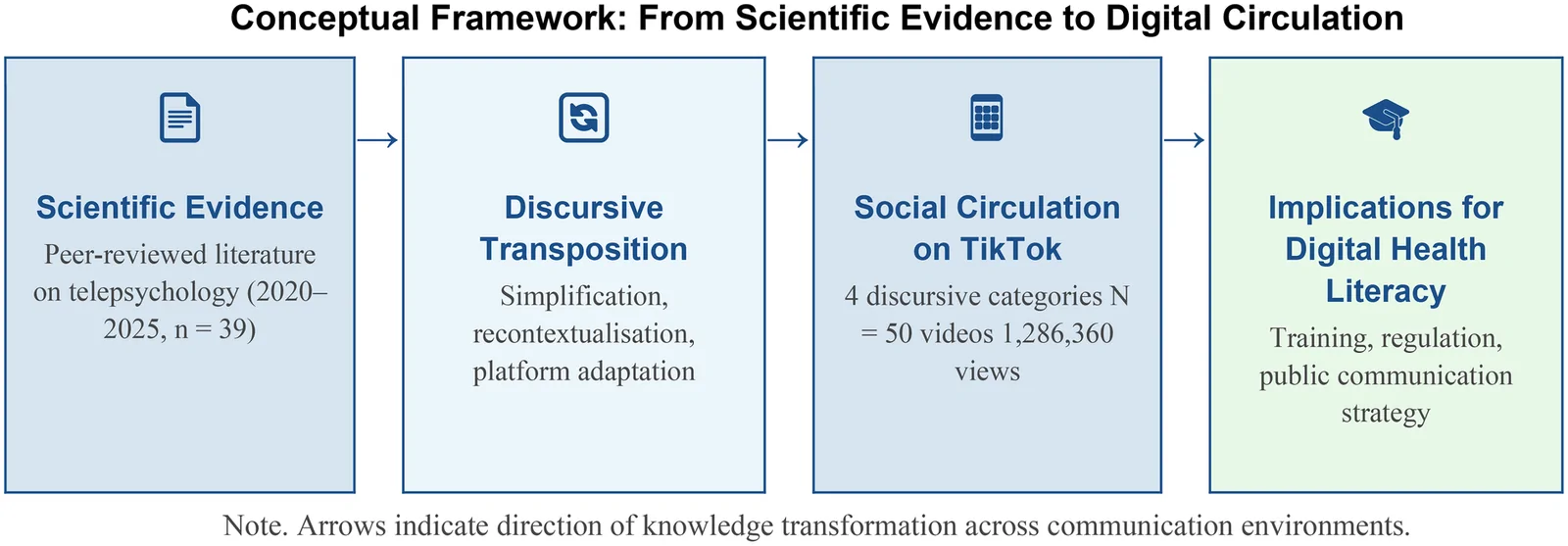
PRISMA-ScR flow diagram illustrating the study selection process for the scoping review (47, 48). Full extraction matrix available in OSF repository.

### 2.3 Tiktok content analysis

To examine the communicative representation of telepsychology, an exploratory analysis of TikTok content was conducted. TikTok was selected for its high penetration among young Spanish-speaking users and the rapid expansion of mental health content on the platform (21). A purposive non-probabilistic sampling strategy was employed (36), using the search terms “telepsicología,” “terapia online,” “psicología virtual,” and “consulta psicológica en línea.”

To reduce algorithmic personalization bias, searches were conducted from a newly created TikTok account with no prior interaction, viewing, or following history, configured in Colombia—the primary institutional affiliation of the research team. TikTok's algorithm applies geolocalised content logic; holding the account's geographic configuration constant across all search sessions ensured consistent retrieval conditions. Videos were included if published in Spanish, containing discursive content explicitly related to telepsychology, exceeding 10,000 views at retrieval (an operational indicator of high platform visibility), and publicly accessible during the study period (December 2024–February 2025). Purely promotional content, duplicated videos, and content lacking discursive relevance to telepsychology were excluded. The final corpus comprised *N* = 50 videos accumulating 1,286,360 total views; approximately 20% of videos concentrated 62% of total views, consistent with platform virality dynamics.

All analyzed videos were publicly accessible at the time of data collection. No automated scraping software, application programming interfaces (APIs), or restricted-access data sources were used. Videos were identified through manual searches using the platform's native search functionality under standardized retrieval conditions. Data collection and analysis were conducted in accordance with TikTok's publicly available Terms of Use and applicable institutional research standards.

### 2.4 Thematic analysis procedures

Thematic analysis followed an inductive strategy inspired by Braun and Clarke's reflexive thematic analysis procedures (37). The analytical process involved three stages: (1) open coding of relevant discursive units; (2) axial grouping of codes into preliminary categories according to semantic similarity and discursive function; and (3) semantic consolidation of final thematic categories through reflexive comparison, verifying internal coherence, conceptual differentiation, and interpretive consistency. Thematic saturation was operationalized as the absence of substantially new categories across three consecutive coding cycles, reached at approximately video 35 (36). Two researchers conducted coding independently; Cohen's kappa calculated on the full corpus of 50 videos yielded *κ* = 0.78 (*p* < .001), indicating substantial agreement (38). Discrepancies were resolved through reflexive analytical discussion until interpretive consensus was achieved.

Since the study employed an inductive qualitative analytical approach rather than a standardized measurement instrument, conventional psychometric validation procedures were not applicable. Analytical trustworthiness was strengthened through independent coding by two researchers, assessment of inter-rater agreement (Cohen's *κ* = 0.78), reflexive discussion of discrepancies, and consensus-based thematic refinement.

### 2.5 Exploratory corpus-descriptive indicators (ICST system)

To facilitate interpretive comparison between scientific representations of telepsychology and the digital circulation patterns observed in the corpus, three exploratory heuristic indicators were computed from thematic frequencies. These are not validated psychometric instruments or inferential tools; they are descriptive ratios and proportions intended to provide a shared quantitative language for comparative interpretation and to guide future studies. Interpretation must remain exclusively referential to the analyzed corpus. Let: n₁ = simplified-definition content (*n* = 16); n₂ = subjectivist experiential content (*n* = 16); n₃ = professional legitimation content (*n* = 10); n₄ = technical language reinterpretation (*n* = 8); *N* = 50. The three indicators are presented in Table 1. These exploratory corpus-descriptive indicators were developed exclusively to facilitate transparent within-corpus comparisons between scientific representations and digital discourse. They do not constitute validated psychometric instruments, epidemiological measures, predictive models, or inferential statistics, and their interpretation should remain strictly limited to the analyzed corpus.

**Table 1:** Exploratory corpus-descriptive indicators (ICST system). Values Computed on the Analyzed TikTok Corpus (*N* = 50 Videos).

Descriptor	Full name	Formula	Value	Corpus-specific interpretation
TGI	Telepsychology Gap Index	1−(n_leg/N)	0.80	80% of corpus videos lacked scientific legitimation or regulatory reference
DTLI	Digital Telepsychology Legitimacy Index	(n_leg + n_tec)/N	0.36	Only ∼1 in 3 videos incorporated clinical, regulatory, or formative grounding
SCI	Simplified Circulation Index	(n_simp + n_sub)/(n_leg + n_tec)	1.78	Simplified/experiential content outnumbered evidence-grounded content by ∼2:1

TGI, telepsychology gap index; DTLI, digital telepsychology legitimacy index; SCI, simplified circulation index. All are exploratory corpus-descriptive indicators, not validated psychometric instruments. Source: authors' own elaboration (2025–2026).

### 2.6 Ethical considerations

The documentary review was limited to publicly available published scientific literature. TikTok content analysis was conducted on publicly accessible material on an open platform, with no intervention or contact with content creators. No sensitive personal data were collected and no individual users were identified; analysis was restricted to general discursive patterns. As the study did not involve direct human participants or identifiable data, institutional ethics committee approval was not required, in accordance with CIOMS (2016) guidelines for observational research in public digital environments.

## 3 Results

### 3.1 Scientific evidence on telepsychology (2020–2025)

The 39 included publications confirm the rapid consolidation of telepsychology as a research object following the COVID-19 pandemic. The literature converges on validating its clinical viability (1, 2) and reporting efficacy levels equivalent to in-person delivery (12–15), with documented benefits in geographical accessibility, stigma reduction, and treatment continuity (5–8, 17). Concerns persist regarding therapeutic alliance adaptation, information security, and ethical-legal standards (2, 20). Regional methodological heterogeneity was observed: longitudinal designs and systematic reviews predominate in Europe and the United States, while Latin American contributions primarily employ exploratory and descriptive designs, evidencing a gap in empirical robustness (19, 39). Scientific output clusters across four research lines: (a) clinical—comparative validation and efficacy; (b) ethical and professional—competencies and modality perceptions; (c) legal-regulatory—governance frameworks; and (d) technical—security standards and platform functionality. Table 2 summarises the distribution across these dimensions.

**Table 2:** Distribution of included studies across research dimensions (scoping review, *n* = 39).

Research Dimension	n	%	Geographic Focus	Predominant Design
Clinical efficacy & validation	16	41%	US, Europe, Australia	RCT, systematic review, meta-analysis
Ethical & professional competencies	10	26%	Latin America, Spain	Exploratory, descriptive, survey
Legal & regulatory frameworks	7	18%	Europe, US	Policy analysis, expert consensus
Technical standards & security	6	15%	International	Technical review, implementation study
Total	39	100%	—	—

Studies classified by primary analytical dimension; some addressed more than one dimension and were assigned to their dominant focus. Geographic classification based on first-author institutional affiliation.

### 3.2 Thematic analysis of the tikTok corpus (*N* = 50)

Inductive thematic analysis identified four recurrent discursive categories in the corpus, reflecting patterns in how telepsychology is communicatively represented within highly viewed TikTok content. Table 3 presents their frequencies and characteristics; Figure 2 displays their proportional distribution.

**Table 3:** Thematic categories identified in the tikTok corpus on telepsychology (*N* = 50 videos).

Category	Description	n	%	Interpretive note
1. Simplified definitions of telepsychology	Frames telepsychology as convenient and immediately accessible; omits therapeutic framing, ethical protocols, clinical assessment, and professional regulation	16	32%	Highest visibility; avg. views exceed corpus mean
2. Subjective and experiential narratives	Personal testimonials emphasizing emotional relief, self-discovery, and subjective transformation; implicit validation through resonance rather than evidence	16	32%	Highest engagement (comments, shares, emotional reactions)
3. Professional legitimacy references	Explicit reference to credentials, ethical standards, evidence-based interventions, or institutional frameworks; accessibility remains platform-adapted	10	20%	Primarily licensed practitioners; lower avg. interaction
4. Reinterpretation of technical language	Clinical constructs (anxiety, trauma, burnout, attachment) adapted through colloquial language and individualized storytelling for rapid comprehension	8	16%	Risk of semantic drift; not systematic misinformation
Total	—	50	100%	*κ* = 0.78, *p* < .001
				

64% of corpus (n=32) corresponded to Categories 1 and 2. Inter-rater reliability: Cohen's *κ*=0.78, p <.001, calculated on full corpus (*N*=50 videos).

**Figure 2:**
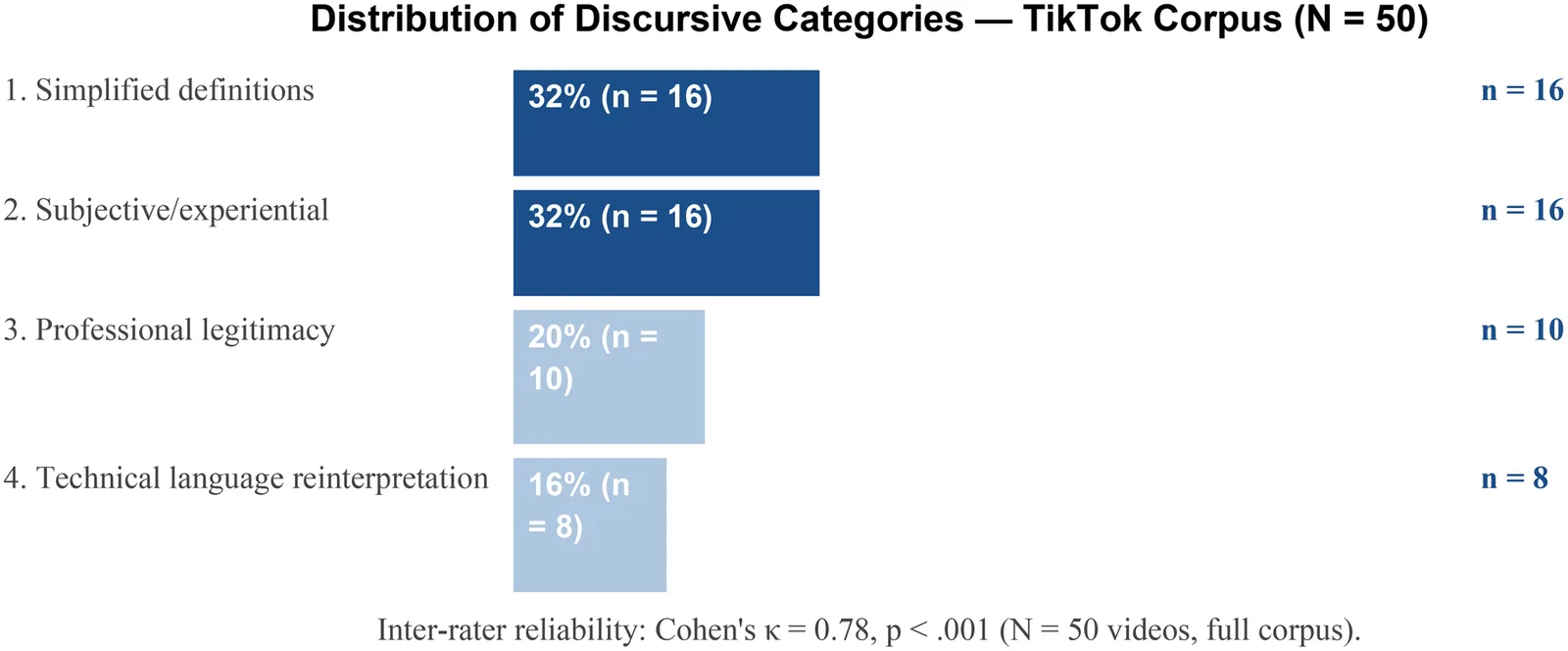
Proportional distribution of discursive categories identified in the analyzed tikTok corpus (*N* = 50 videos). Bars represent percentage of corpus; *n* values indicate absolute frequency.

Category 1 (Simplified definitions, 32%) framed telepsychology primarily as immediate, convenient, and emotionally accessible, omitting discussion of therapeutic framing, ethical procedures, clinical assessment, and professional regulation. Category 2 (Subjective and experiential narratives, 32%) emphasized emotional relief, personal transformation, and self-discovery through testimonial formats; experiential discourse functioned as an implicit validation mechanism replacing scientific reference. Category 3 (Professional legitimacy references, 20%) included explicit credentials, ethical standards, and evidence-based language, though even these contents remained shaped by platform dynamics favouring brevity and emotional engagement. Category 4 (Reinterpretation of technical language, 16%) involved colloquial adaptation of clinical constructs—anxiety, trauma, burnout, attachment—optimized for rapid comprehension and algorithmic visibility, without constituting systematic misinformation.

### 3.3 Exploratory corpus-descriptive indicators (ICST)

Three heuristic indicators were computed to quantify within-corpus patterns. The Telepsychology Gap Index (TGI = 0.80) indicates that 80% of corpus videos lacked any element of scientific legitimation or regulatory reference. The Digital Telepsychology Legitimacy Index (DTLI = 0.36) indicates that approximately one in three videos incorporated clinical, regulatory, or formative foundations. The Simplified Circulation Index (SCI = 1.78) indicates that simplified/experiential content outnumbered technically grounded content by a ratio of approximately 2:1. These values are corpus-specific and exploratory; they are not generalisable and should not be interpreted as validated metrics of misinformation prevalence. Table 1 presents the indicators with their formulae and interpretations; Figure 3 illustrates their relative values.

**Figure 3:**
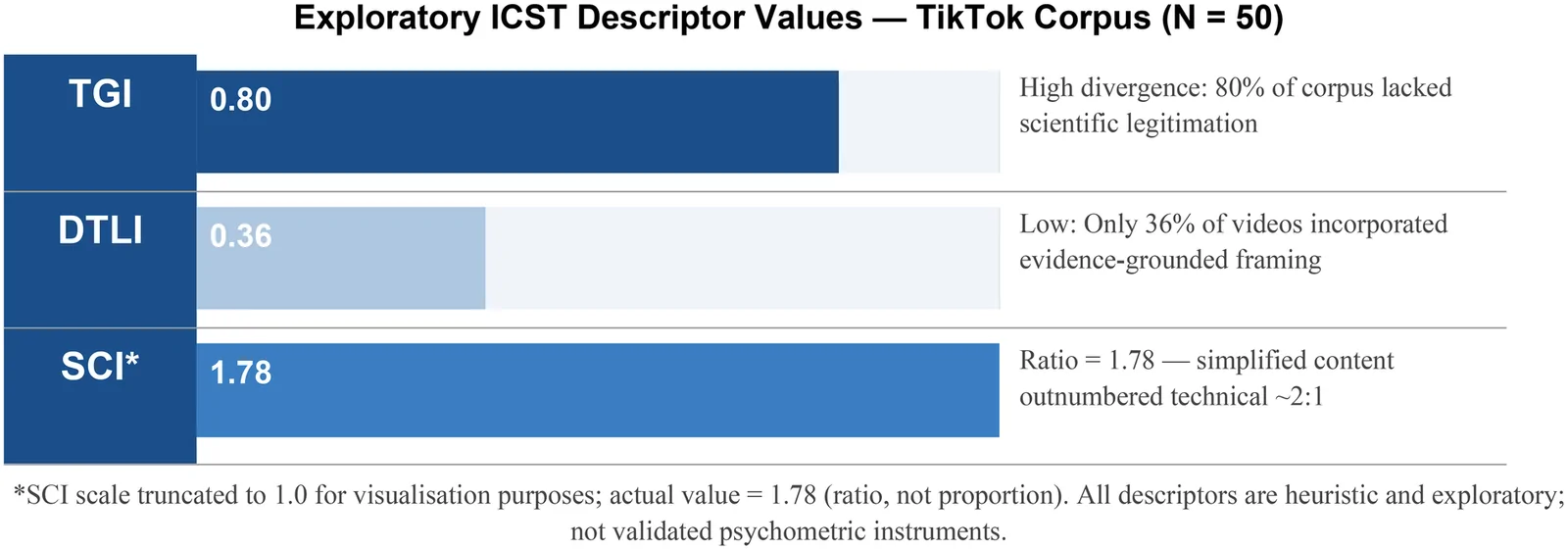
Exploratory ICST indicator values computed from thematic frequencies in the analyzed tikTok corpus (*N* = 50). *SCI bar truncated to proportion scale for visualization; actual ratio value = 1.78. All indicators are heuristic and exploratory.

### 3.4 Integrative comparison: scientific evidence vs. digital circulation

Table 4 sssynthesizes the structural differences identified between the reviewed scientific literature and the digital representations observed in the TikTok corpus, organized across eight analytical dimensions.

**Table 4:** Comparative matrix of discursive transposition: scientific production (*n* = 39) versus social appropriation in the analyzed tikTok corpus (*N* = 50).

Dimension	Scientific literature (*n* = 39)	TikTok corpus (*N* = 50)	Observed divergence pattern
Validation source	Empirical evidence, systematic reviews, regulatory frameworks	Personal experience and testimonials (Cat. 2, 32%); convenience narratives (Cat. 1, 32%)	Personal narrative displaces clinical authority
Conceptual framing	Technical rigor; structured clinical processes and exclusion criteria	Functional equivalence ('therapy by video call'); instrumental accessibility	Clinical depth reduced to technological convenience
Efficacy criteria	Validated symptom scales, longitudinal outcomes, therapeutic alliance	Immediate satisfaction, speed, and emotional comfort	User convenience prioritized over therapeutic effectiveness
Ethical dimension	Informed consent, encryption standards, remote crisis protocols	Occasional mentions; motivational and emotional language dominates	Ethical rigor softened in favour of communicative proximity
Professional role	Specialised training, digital competencies, clinical supervision	Content creator, wellness guide, emotionally accessible advisor	Partially informalised professional figure
Language register	Technical-academic; evidence-based terminology	Colloquial, short-form video, persuasive narrative (Cat. 4, 16%)	Adapted for viral circulation and algorithmic visibility
Identified risks	Digital divide, data security, crisis management protocols	Low problematisation of application conditions or access barriers	Structural risks underrepresented in digital discourse
Discursive logic	Clinical precision and contextualised interpretation	Engagement optimisation; emotional resonance; immediacy	Platform structure shapes knowledge circulation criteria

Observed patterns describe tendencies within the analyzed corpus and do not constitute generalisable claims about all digital content on telepsychology. TikTok column entries reference category frequencies in Table 2 where applicable.

## 4 Discussion

### 4.1 Telepsychology as a consolidated modality: evidence and methodological tensions

The scoping review confirms that telepsychology has transitioned from a contingent pandemic response to constitute a structurally relevant modality of mental health care, backed by comparative efficacy evidence and growing professional acceptance (12, 13, 16). Its legitimacy is reinforced across geographically diverse contexts (1, 6, 14). However, regional methodological heterogeneity—with longitudinal and experimental designs concentrated in the Global North and exploratory studies predominating in Latin America—indicates that the field's empirical consolidation remains uneven (19, 39). This gap reinforces the importance of investing in regionally grounded research and normative standardisation in contexts such as Colombia and Chile, where telepsychology's regulatory architecture is still developing.

### 4.2 Digital resignification: from clinical rigour to perceived convenience

Within the analyzed corpus, digital content evidences a predominantly functional representation of telepsychology centred on accessibility and immediate experience, in contrast with the scientific literature's orientation toward validating efficacy, delimiting competencies, and consolidating regulatory frameworks (18–20). Following Zenone et al. (22), this pattern reflects a communicative logic—rather than systematic deception—governing knowledge circulation on digital platforms: content is optimised for engagement, not for technical precision. Nevertheless, when such content becomes the primary information source for broad population sectors, its effect on therapeutic expectations and public understanding of the clinical process may be considerable.

The findings are consistent with prior documentation of analogous dynamics in specific mental health conditions. Lorenzo-Luaces et al. (27) found TikTok content on cognitive-behavioural therapy prioritises experiential over technical elements; Turuba et al. (28) identified similar simplifications in depression content; Yeung et al. (29) documented reductionist ADHD representations.

### 4.3 The ICST indicators and their heuristic value

The three corpus-descriptive indicators allow exploratory quantification of observed circulation patterns. The TGI (0.80) indicates that four in five corpus videos lacked scientific legitimation elements. The DTLI (0.36) indicates that barely one third incorporated evidence-grounded framing. The SCI (1.78) evidences that simplified content predominated over technically grounded content by approximately 2:1. These are corpus-specific, exploratory values—not validated metrics. Their purpose is to facilitate interpretive comparison within the corpus and to support the design of future studies with larger samples, computational methods, and cross-platform comparative designs.

### 4.4 Implications for training, regulation, and public communication

Taken together, the present findings suggest three convergent implications for education, professional regulation, and public communication (Figure 4). Colón Llamas et al. (40) and Sáenz et al. (18) have emphasised the need to incorporate telepsychology competencies into training curricula; the present findings extend this imperative toward the capacity to communicate clinical practice responsibly in the digital spaces where users encounter it first. In regulatory terms, existing frameworks—APA (9), EFPA (10), PAHO (4)—address clinical practice but not professional communication on social media; the European Digital Services Act (2022) and its Latin American counterparts under development offer starting points for standards of informational responsibility in digital mental health. In public communication terms, McCashin and Murphy (23) and Merga (34) indicate that Digital health literacy interventions on platforms such as TikTok require active professional participation as critical knowledge mediators (Dadaczynski et al., 2021; Merga, 2024) (26). The capacity to produce simultaneously rigorous and communicatively effective digital narratives is an emerging twenty-first-century competency requiring specific training and institutional recognition (41–46).

**Figure 4:**
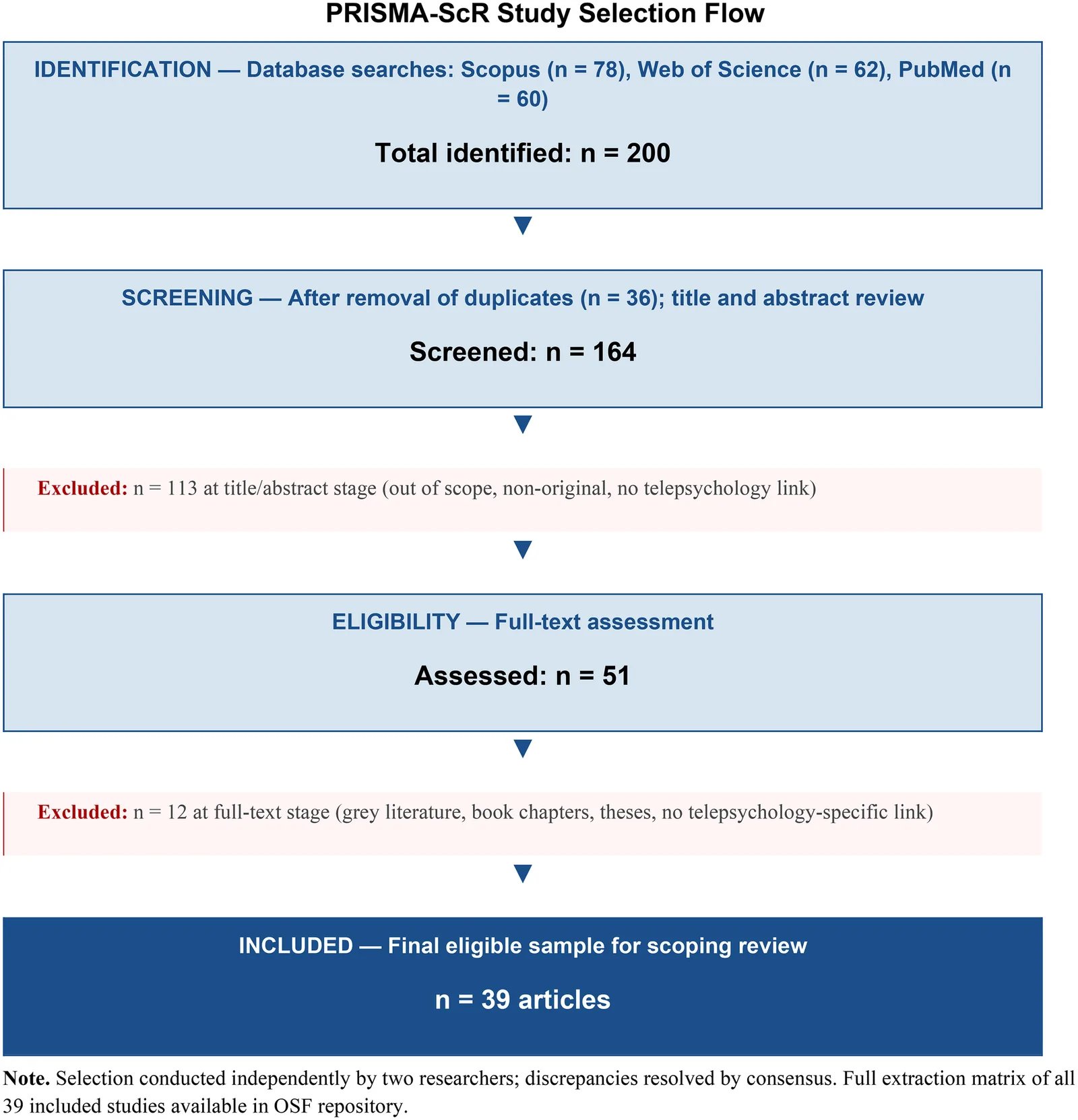
Conceptual framework illustrating the relationship between scientific evidence on telepsychology, digital discursive transposition, social circulation dynamics on tikTok, and implications for digital mental health literacy.

### 4.5 Limitations

The findings carry generalisability constraints arising from: (1) non-probability sampling from a single platform; (2) the bounded temporal window (December 2024–February 2025), which may reflect seasonal effects on content production and algorithmic prioritisation; (3) TikTok's geolocalised algorithmic logic—despite the implemented control (newly created account configured in Colombia)—cannot be fully eliminated; (4) the exclusive focus on Spanish-language content; and (5) the absence of external validation of the ICST indicators. Extending these findings to other platforms (Instagram, YouTube, Facebook), linguistic regions, and temporal periods constitutes a priority research agenda, as does computational analysis of larger multilingual corpora. Additionally, TikTok recommendation algorithms evolve continuously over time. Consequently, identical search strategies implemented at different time points may retrieve partially different corpora despite equivalent search procedures. The present findings should therefore be interpreted as a time-specific analytical snapshot rather than a permanent representation of platform-wide discourse

## 5 Conclusions

Telepsychology has consolidated as a structurally relevant modality within contemporary digital mental health care, supported by evidence of clinical efficacy comparable to in-person delivery. At the same time, the patterns observed in the analyzed TikTok corpus indicate that its representation in digital ecosystems operates under a markedly different discursive logic: communicative accessibility and immediate subjective experience displace clinical validation, ethical standards, and professional competencies as dominant evaluative criteria.

The exploratory corpus-descriptive indicators (TGI = 0.80; DTLI = 0.36; SCI = 1.78) quantify this gap within the corpus and provide a methodological starting point for future comparative research. Their principal value lies not in the absolute figures—which are sample- and platform-dependent—but in the comparative logic they instantiate and the research agenda they orient.

The sustainable consolidation of telepsychology depends on three convergent pillars: (1) empirical robustness, with longitudinal and standardised research especially in Latin American contexts; (2) updated normative frameworks that address professional communication in digital environments, not only clinical practice; and (3) systematic digital mental health literacy strategies that link academic production with the social circulation dynamics of knowledge on high-reach platforms. The figure of the psychologist as a critical mediator in the digital ecosystem emerges as a twenty-first-century professional competency requiring specific training, institutional recognition, and continued theoretical development.

Beyond describing a divergence between scientific evidence and platform circulation, the study highlights that telepsychology is being socially interpreted under communicative conditions that privilege immediacy, emotional resonance, and accessibility over technical precision. This observation is relevant not only for digital health communication research, but also for the broader consolidation of telepsychology as a legitimate mode of care, since public expectations about online therapy may increasingly be shaped before any formal clinical contact occurs. In this sense, strengthening digital mental health literacy should not be understood as an exclusively individual task. It also requires institutional, educational, and professional strategies capable of translating scientific knowledge into culturally situated and communicatively effective formats across digital platforms. Future work should therefore connect comparative platform research, culturally sensitive analysis, and intervention-oriented approaches that help bridge the gap between academic evidence and the everyday social circulation of mental health content.

## Data Availability

The original contributions presented in the study are included in the article/Supplementary Material, further inquiries can be directed to the corresponding author.
