# Supplementary material for: Telepsychology and digital mental health communication: an exploratory mixed-methods study of tikTok content
Source: Front Digit Health. 2026 Sep 15;8:1900544. doi: 10.3389/fdgth.2026.1900544 (PMC13621891; doi:10.3389/fdgth.2026.1900544)

Supplementary Figure S1

PRISMA-ScR flow diagram illustrating the study selection process for the scoping review.

This supplementary material accompanies the manuscript "Telepsychology and Digital Mental Health Communication: An Exploratory Mixed-Methods Study of TikTok Content."

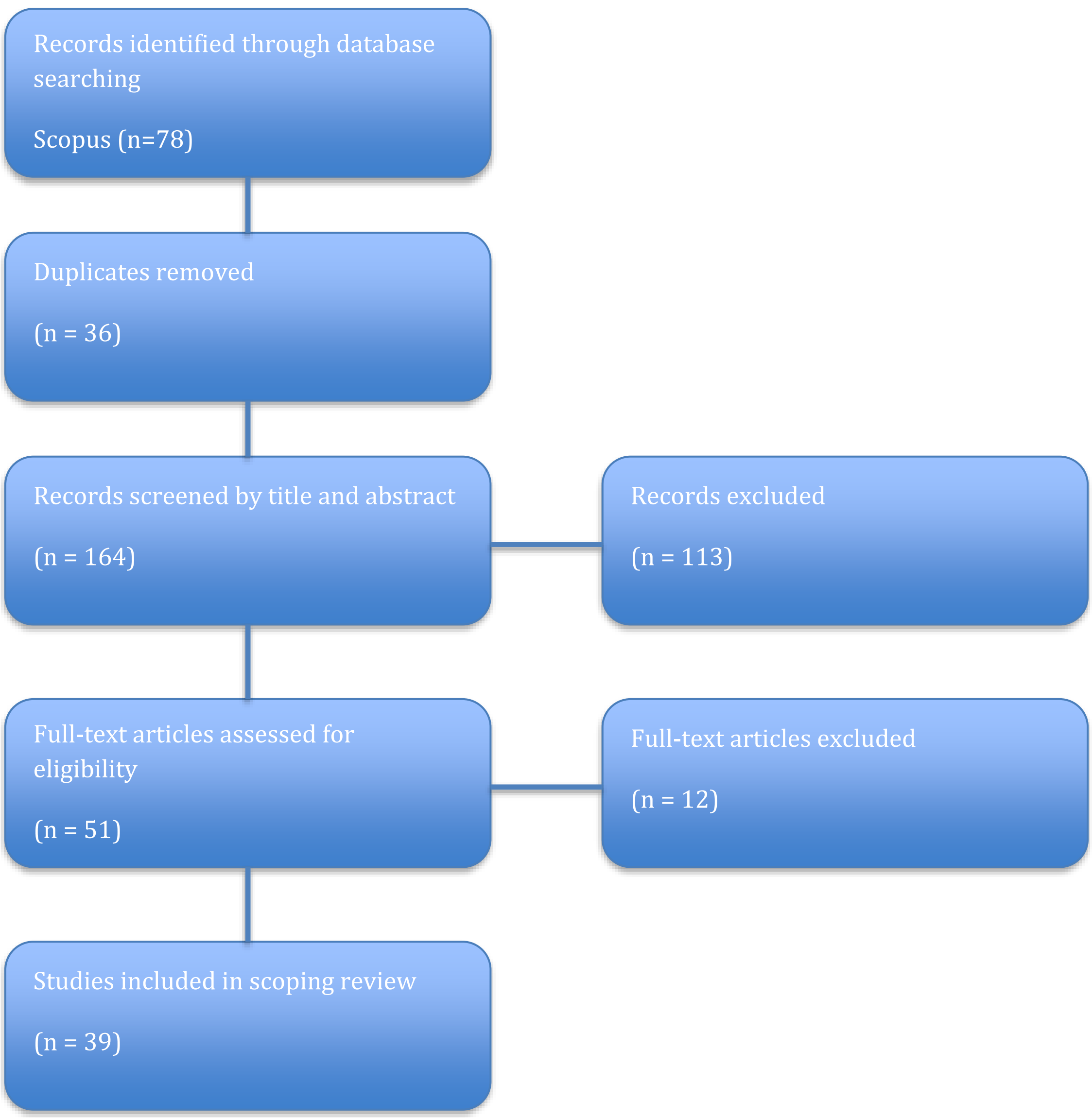

Supplement: Supplementary file 1 [file Image1.pdf]
